# HIF1α is a direct regulator of steroidogenesis in the adrenal gland

**DOI:** 10.1007/s00018-020-03750-1

**Published:** 2021-01-19

**Authors:** Deepika Watts, Johanna Stein, Ana Meneses, Nicole Bechmann, Ales Neuwirth, Denise Kaden, Anja Krüger, Anupam Sinha, Vasileia Ismini Alexaki, Luis Gustavo Perez-Rivas, Stefan Kircher, Antoine Martinez, Marily Theodoropoulou, Graeme Eisenhofer, Mirko Peitzsch, Ali El-Armouche, Triantafyllos Chavakis, Ben Wielockx

**Affiliations:** 1 Institute of Clinical Chemistry and Laboratory Medicine, Technische Universität Dresden, Fetscherstrasse 74, 01307 Dresden, Germany; 2 Department of Experimental Diabetology, German Institute of Human Nutrition Potsdam-Rehbruecke, 14558 Nuthetal, Germany; 3 German Center for Diabetes Research (DZD), 85764 München-Neuherberg, Germany; 4 Medizinische Klinik Und Poliklinik IV, Ludwig-Maximilians-Universität (LMU) München, Munich, Germany; 5 Institute of Pathology, University Würzburg, Würzburg, Germany; 6 Génétique Reproduction and Dévelopement (GReD), Centre National de la Recherche Scientifique (CNRS), INSERM, Université Clermont-Auvergne, Clermont-Ferrand, France; 7 Department of Pharmacology and Toxicology, Medical Faculty, Technische Universität Dresden, 01307 Dresden, Germany

**Keywords:** Hypoxia-inducible factor, Adrenocortical steroids, Oxygen sensors, Cytokines

## Abstract

Endogenous steroid hormones, especially glucocorticoids and mineralocorticoids, derive from the adrenal cortex, and drastic or sustained changes in their circulatory levels affect multiple organ systems. Although hypoxia signaling in steroidogenesis has been suggested, knowledge on the true impact of the HIFs (Hypoxia-Inducible Factors) in the adrenocortical cells of vertebrates is scant. By creating a unique set of transgenic mouse lines, we reveal a prominent role for HIF1α in the synthesis of virtually all steroids in vivo. Specifically, mice deficient in HIF1α in adrenocortical cells displayed enhanced levels of enzymes responsible for steroidogenesis and a cognate increase in circulatory steroid levels. These changes resulted in cytokine alterations and changes in the profile of circulatory mature hematopoietic cells. Conversely, HIF1α overexpression resulted in the opposite phenotype of insufficient steroid production due to impaired transcription of necessary enzymes. Based on these results, we propose HIF1α to be a vital regulator of steroidogenesis as its modulation in adrenocortical cells dramatically impacts hormone synthesis with systemic consequences. In addition, these mice can have potential clinical significances as they may serve as essential tools to understand the pathophysiology of hormone modulations in a number of diseases associated with metabolic syndrome, auto-immunity or even cancer.

**Supplementary Information:**

The online version contains supplementary material available at 10.1007/s00018-020-03750-1.

## Introduction

Steroidogenesis in the adrenal gland is a complex process of sequential enzymatic reactions that convert cholesterol into steroids, including mineralocorticoids and glucocorticoids [1]. While glucocorticoids are regulated by the hypothalamic–pituitary–adrenal axis (HPA axis) and are essential for stress management and immune regulation [2, 3], aldosterone, the primary mineralocorticoid, regulates the balance of water and electrolytes in the body [4]. As steroidogenesis is a tightly regulated process, proper control of adrenal cortex function relies on appropriate endocrine signaling, tissue integrity, and homeostasis [5]. Accordingly, it has been suggested that inappropriately low pO_2_, or hypoxia, can lead to both structural changes in the adrenal cortex and interfere with hormone production [6–10].

Hypoxia-inducible factors (HIFs) are the main transcription factors that are central to cellular adaptation to hypoxia in virtually all cells of our body. The machinery that directly controls HIF activity consists of the HIF-prolyl hydroxylase domain-containing enzymes (PHD-1, -2, -3) (encoded by the *Egln-2*,* -1*,* -3* genes, respectively), which are oxygen sensors that hydroxylate two prolyl residues in the HIFα subunit under normoxic conditions, thereby marking the HIFs for proteasomal degradation. Conversely, oxygen insufficiency renders these PHDs inactive, leading to the binding of the HIF-complex to hypoxia-responsive elements (HRE) in the promoter of multiple genes that ensure oxygen delivery and promote adaptive responses to hypoxia such as hematopoiesis, blood pressure regulation, and energy metabolism (reviewed in [11, 12]). Apart from directly activating hypoxia-responsive genes [13, 14], HIFs also indirectly influence gene expression by interfering with the activity of other transcription factors or systems. Of the most intensively studied HIFα genes, HIF1α has a ubiquitous pattern of expression in all tissues, whereas expression of the paralogue HIF2α is restricted to a selection of cell types including endothelial cells,, liver hepatocytes, epithelial cells of the intestinal lumen, glia cells in the brain and renal erythropoietin-producing cells [15–18].

Recent in vitro and zebrafish studies have revealed a continuous crosstalk between HIF and steroidogenesis pathways, along with potential interference in the production of aldosterone and glucocorticoids [19–22]. Most hypoxia-related findings on steroidogenesis come from granulosa cells, either showing a direct role for HIF1α in regulating Star, the mitochondrial cholesterol transporter [20, 23], or suggesting an inverse role on steroidogenesis [24]. In the adrenocortical carcinoma cell line H295R, hypoxia resulted in downregulation of steroidogenic genes, while downregulation of CYP19A1 was regulated by HIF1 via induction of miR-98 [25]. There is also evidence suggesting a role for the hypoxia pathway in modulating glucocorticoid/glucocorticoid receptor (GR) signaling [26, 27]. Importantly, these observations indicate a possible interplay of HIFs and PHDs in modulating the immune-regulatory actions of the HPA axis. Currently, there is huge interest in the development of HIF inhibitors and HIF stabilizers, and their influence on medicine is expected to become significant in the near future [28]. However, as the role of HIFs/PHDs is both central and manifold with respect to maintaining oxygen homeostasis, a better understanding of the true impact of Hypoxia Pathway Proteins (HPPs) in the complex interplay of different essential physiological and pathological conditions, including in the adrenal cortex, assumes great importance.

We describe the creation of a unique collection of transgenic mouse lines that enables an investigation of the role of HIFα subunits in adrenocortical cells and beyond. Our results point towards a central role for HIF1α in the direct regulation of steroidogenesis in the adrenal gland and consequent changes in circulatory hormone levels. Importantly, chronic exposure of mice to such altered hormone levels eventually led to a dramatic decrease in essential inflammatory cytokines and profound dysregulation of circulatory immune cell profiles.

## Materials and methods

### Mice

All mouse strains were housed under specific pathogen-free (SPF) conditions at the Experimental Centre of the Medical Theoretical Center (MTZ, Technical University of Dresden-University Hospital Carl-Gustav Carus, Dresden, Germany). Experiments were performed with male and female mice aged between 8 and 16 weeks. Akr1b7:cre-*Phd2*/*Hif1*^*ff/ff*^ (P2H1) or Akr1b7:cre-*Phd2/Phd3*^*ff/ff*^ (P2P3) lines were generated by crossing Akr1b7:cre mice [29] to *Phd2*^*f/f*^,* Hif1α*^*f/f*^ or *Phd2*^*f/f*^*; Phd3*^*f*/f^ as previously reported by us [30], and/or the reporter strain mTmG [31]. All mice described in this report were born in normal Mendelian ratios and were bred on a C57BL/6J background (backcrossed at least 9 times). For each experiment, transgenic mice were compared to littermate controls. Mice were genotyped using primers described in Online Resource 1. Both genders were used in similar amounts and no significant differences between the genders of the same genotype were observed for any of the performed analysis within this study.

Histological analysis of the adrenal gland of Akr1b7:cre-mTmG^f/f^ reporter mice revealed zonal variation in the penetrance of cre-recombinase activity in the adrenal cortex of all individual mice (GFP^+^ staining). Peripheral blood was drawn from mice by retro-orbital sinus puncture using heparinized micro hematocrit capillaries (VWR, Darmstadt, Germany) and plasma separated and stored at − 80 °C until further analysis. Mice were killed by cervical dislocation and adrenals were isolated, snap frozen in liquid nitrogen, and stored at − 80 °C for hormone analysis or gene expression analysis. All mice were bred and maintained in accordance with facility guidelines on animal welfare and with protocols approved by the Landesdirektion Sachsen, Germany.

### Blood analysis

White blood cells, including neutrophils, eosinophils and lymphocytes were measured in whole blood using a Sysmex automated blood cell counter (Sysmex 117 XE-5000)) [32].

### Hormone detection

Adrenal glands were incubated in disruption buffer (component of Invitrogen™ Paris™ Kit, AM 1921, ThermoFisher Scientific, Dreieich, Germany) for 15 min at 4 °C, homogenized in a tissue grinder, followed by incubation for 15 min on ice, centrifugation and supernatant collection (N.B. and M.P. unpublished results). *Adrenal steroid hormones* were determined by LC–MS/MS as described elsewhere [33]. *Catecholamines*, norepinephrine, epinephrine, and dopamine were measured by high-pressure liquid chromatography (HPLC) coupled with electrochemical detection, as previously described [34]. The hormones were measured as ng per µg of total adrenal gland protein, ranging from ~ 4 to ~ 50 ng/adrenal for progesterone; ~ 10– ~ 1500 ng/adrenal for corticosterone, and ~ 3– ~ 27 ng/adrenal for aldosterone. All concentrations were normalized to the average value of WTs for every independent experiment; and the average WT value was set as 1.

### RNA extraction and qPCRs

RNA from adrenal glands and sorted cells was isolated using the RNA Easy Plus micro kit (Qiagen) (Cat. # 74034Qiagen). cDNA synthesis was performed using the iScript cDNA Synthesis Kit (BIO-RAD, Feldkirchen, Germany). Gene expression levels were determined by performing quantitative real-time PCR using the ‘Ssofast Evagreen Supermix’ (BIO-RAD, Feldkirchen, Germany). Sequences of primers used are provided in Online Resource 2. Expression levels of genes were determined using the Real-Time PCR Detection System-CFX384 (BIO-RAD, Feldkirchen, Germany). All mRNA expression levels were calculated relative to β2M or EF2 housekeeping genes and were normalized using the ddCt method. Relative gene expression was calculated using the 2(− ddCt) method, where ddCT was calculated by subtracting the average WT dCT from dCT of all samples individually.

### Immunohistochemistry and immunofluorescence

For preparation of paraffin sections, adrenal glands were isolated, incubated in 4% formaldehyde at 4 °C overnight, dehydrated, embedded in paraffin and cut into 5 µm sections using a microtome. Sections were rehydrated and subjected to hematoxylin and eosin staining (H&E). For frozen sections, adrenal glands were embedded in O.C.T Tissue-Tek (A. Hartenstein GmbH, Würzburg, Germany) and stored at − 20 °C. For H&E staining of frozen sections (7 µm), samples were first fixed in cold acetone before staining. For immunofluorescence, sections were fixed in cold acetone, air-dried, washed with phosphate-buffered saline containing 0.1% Tween-20, blocked with 5% normal goat serum followed by primary antibody staining (CD31/PECAM—1:500 [35]) or GFP Polyclonal (Antibody ThermoFisher Scientific—1:200) overnight at 4 °C and subsequent secondary antibody staining. After counterstaining with DAPI, slides were mounted in fluorescent mounting medium and stored at 4 °C until analysis.

### Microscopy

Both bright-field and fluorescent images were acquired on an ApoTome II Colibri (Carl Zeiss, Jena, Germany). Images were analyzed using either Zen software (Carl Zeiss, Jena, Germany) or Fiji (ImageJ distribution 1.52 K). Fiji was used to quantify lipid droplet sizes and amount of CD31 staining per area.

### Meso Scale Discovery

Plasma was collected from whole blood (3000 RPM for 10 min at 4 ℃). Meso Scale Discovery (MSD, Rockville, Maryland) was used for quantitative determination of the cytokines (IL-1β, IL-4, IL-5, IL-6, KC/GRO, IL-10, and TNF-α) using 50 µl of plasma in the Proinflammatory Panel 1 (mouse) V-PLEX Kit and MSD plate reader (QuickPlex SQ 120). Cytokine concentrations were calculated by converting the measured MSD signal to pg/ml using a standard. All values below blank (control) were considered as zero. Finally, all cytokine concentrations in individual transgenic mice were normalized to the average value of WTs for every independent experiment; and the average WT value was set as 1.

### Next-generation sequencing

For RNAseq analysis, adrenal glands from Akr1b7:cre-*Phd2/Hif1*/*mTmG*^fff/fff^ and Akr1b7:cre-mTmG^f/f^ (control) mice were isolated directly into the lysis buffer of the RNeasy Plus Micro Kit, RNA was isolated according to manufacturer’s instructions, and SmartSeq2 sequencing was performed [RNAseq data are available at GEO (GSE154032)]. Flow cytometry and cell sorting were performed as described previously [36].

### Read quantification

Kallisto v0.43 was first used to generate an index file from the transcript file, which can be downloaded from:ftp://ftp.ebi.ac.uk/pub/databases/gencode/Gencode_mouse/release_M12/gencode.vM12.transcripts.fa.gz. Kallisto v0.43 was then run on all the fastq files using parameters “quant –single -l 75 -s 5 -b 100” to quantify reads for the genes.

### Differential gene expression quantification

Complete cDNA sleuth v0.30.0 (an R package) was used to evaluate differential expression. The command “sleuth_prep” was run with parameter “gene_mode = TRUE”. Two separate error models were fit using “sleuth_fit” wherein the first was a “full” model with gender and experimental condition as covariates, while the second was a “reduced” model with only gender as the covariate. “sleuth_lrt” (Likelihood Ratio Test) was used to evaluate differential gene expression by comparing the full model and the reduced model.

### Statistical analyses

All data are presented as mean ± SEM. Data (WT control versus transgenic line) were analyzed using the Mann–Whitney *U* test, unpaired *t* test with Welch’s correction as appropriate (after testing for normality with the F test) or as indicated in the text. All statistical analyses were performed using GraphPad Prism v7.02 or higher for Windows (GraphPad Software, La Jolla California USA, www.graphpad.com); “n” in the figure legends denotes individual samples.

## Results

### A new mouse model to study the effects of HIFα in the adrenal cortex

**Fig. 1:**
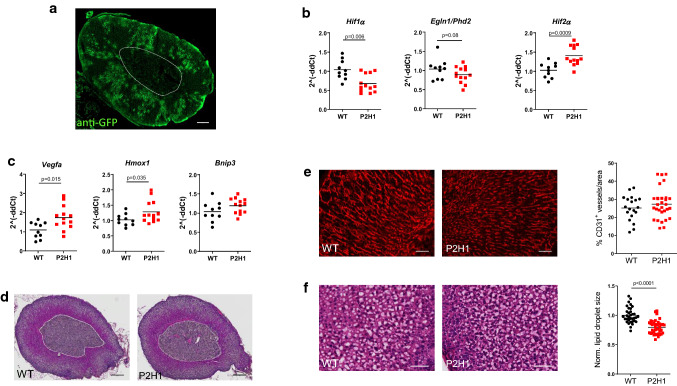
Characterization of the Akr1b7:cre-P2H1^ff/ff^ mouse line with cortex-specific targeting of hypoxia pathway proteins. **a** Representative immunofluorescent image of anti-GFP stained (GFP +) area in the adrenal cortex of the Akr1b7:cre-mTmG mouse line. Region enclosed within the white dotted line represents the medulla and it demarcates the medulla from the cortex (scale bar, 100 μm). **b** qPCR-based mRNA expression analysis of *Hif1α*,* Phd2 and Hif2α* in entire adrenal tissue from P2H1 mice and WT littermates (*n* = 10–13). Relative gene expression was calculated using the 2^(^−^ddCt) method. The graphs represent data from 2 independent experiments. **c** Relative gene expression analysis using mRNA from the entire adrenal tissue in P2H1 mice and their WT counterparts (*n* = 10–13). All graphs represent data from 2 independent experiments. **d** Representative images (magnification 20x) of paraffin sections of adrenal glands (H&E) from 8-week-old WT and P2H1 mice (scale bars represent 100 μm). **e** Representative immunofluorescent images of CD31^+^ endothelial cell staining in adrenal gland sections from WT and P2H1 mice (scale bars represent 50 μm). Graph in the right-side panel represents quantification of CD31^+^ area as a fraction of total tissue area. Each data point represents a single measurement of the cortical area in the adrenal gland (collection of *n*= 6 vs 11 individual mice). **f** Representative images of cryosections of WT and P2H1 adrenal glands (H&E) (scale bars represent 50 μm). Graph in the right-side panel represents the normalized average size of an individual lipid droplet per section of adrenal gland tissue in WT versus P2H1 mice. Measurements were made from 6 sections per mouse. (*n* = 8 individual adrenals per genotype). The graphs in **e** and **f** are representative of 2 independent experiments

We took advantage of the adrenal cortex-specific *Akr1b7*:cre recombinase mouse line (no gonadal expression) [30] to investigate the effects of adrenocortical HIF1α and/or HIF2α on the structure and functions of the adrenal gland. First, when combined with the mTmG reporter strain [31], we demonstrate partial targeting of adrenocortical cells as shown previously (Fig. 1a) [30]. We then combined this cre-line to locally knock-out PHD2, the HIFα’s direct regulator, together with HIF1α; generating the *Akr1b7:cre*-*Phd2/Hif1*^*ff/ff*^ mouse line (henceforth designated P2H1). Importantly, qPCR analysis using mRNA from whole adrenal glands already revealed significant reduction of *Hif1α*, a clear tendency in reduced *Phd2* expression and a substantial increase of *Hif2α* mRNA when compared to glands from WT littermates (Fig. 1b). Furthermore, we explored the expression profile of a number of downstream genes known to be transactivated by HIF2α [37–39] and found a significant increase in *Vegfa*,* Hmox1*, and a trend in *Bnip3* levels (Fig. 1c). Taken together, P2H1 mice exhibit markedly opposite expression levels of *Hif1α* and *Hif2α* confined to adrenocortical cells, which we were even able to define in whole adrenal glands.

### Morphological changes in the adrenal cortex of P2H1 mice

To evaluate the impact of changes in HIF1α and/or HIF2α activity in adrenocortical cells, we analyzed adrenal gland morphology using H&E staining on paraffin sections but found no differences between P2H1 mice and WT littermates in the structure of the adrenal gland, especially, at the side of the cortex of P2H1 mice in comparison to WT littermates (Fig. 1d). As we detected a significant increase in *Vegfa* in the adrenal glands of P2H1 mice, we used CD31 staining to quantify endothelial cells but detected no significant differences between P2H1 and WT mice (Fig. 1e). Remarkably, H&E staining on cryosections of P2H1 adrenal glands revealed significantly smaller lipid droplets in the adrenocortical cells (Fig. 1f), an effect that is reported to be correlated with greater conversion of cholesterol into pregnenolone [10].

### Modulation of HPPs in the adrenal cortex enhances synthesis and circulatory levels of steroid hormones

**Fig. 2:**
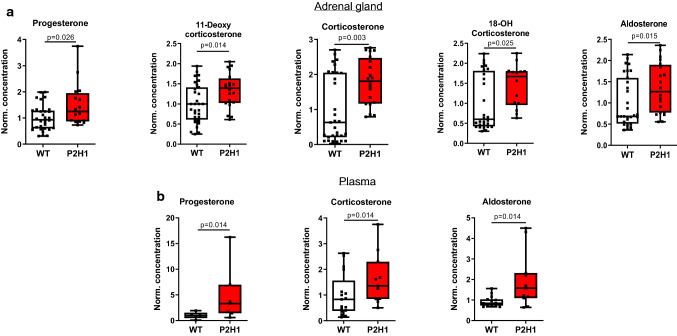
Adrenal cortex-specific loss of PHD2 and HIF1 leads to enhanced steroidogenesis in P2H1 mice. **a** Box and whisker plots showing steroid hormone measurements in adrenal glands from WT mice and compared to littermate P2H1 mice (*n* = 20–31 individual adrenal glands). **b** Box and whisker plots showing steroid hormone measurements in the plasma of individual mice (*n* = 5–17). All data were normalized to the average value of WT mice and graphs represent results of pooled data of at least 3 independent experiments

Next, to verify if the observed changes in lipid droplets indeed led to changes in steroidogenesis, we quantified steroid hormones and their precursor levels by LC–MS/MS in the adrenal gland and in plasma. Quantification revealed a significant increase in virtually all the hormones tested in P2H1 adrenal glands compared to WT littermates (Fig. 2a), and importantly, a corresponding increase of progesterone, corticosterone, and aldosterone was found in the plasma (Fig. 2b). These observations clearly indicate that central HPPs have an impact on steroidogenesis in the murine adrenal gland and on circulatory levels of steroid hormones.

### Downstream effects of the chronic increase in the steroidogenesis

Previous reports have stated that glucocorticoids can regulate catecholamine production in the adrenal medulla [40, 41]; therefore, we also measured dopamine, norepinephrine, and epinephrine levels in the samples used to quantify steroid levels (as above). However, we found no difference between P2H1 and WT littermates in any of the catecholamines quantified (Supplementary Fig. S1a). Further, although increased steroid levels often result in other systemic changes, P2H1 mice displayed no difference in serum potassium levels or blood glucose levels compared to WT littermates (Supplementary Fig. S1b, c). Taken together, in contrast to the systemic effects induced by acute and high levels of circulatory cortical hormones (e.g., corticosterone, aldosterone) [3, 4], the P2H1 mice display moderate but chronically enhanced levels of cortical hormones at the described time points.

### Loss of PHD2/HIF1α in adrenocortical cells impacts gene expression related to steroidogenesis

**Fig. 3:**
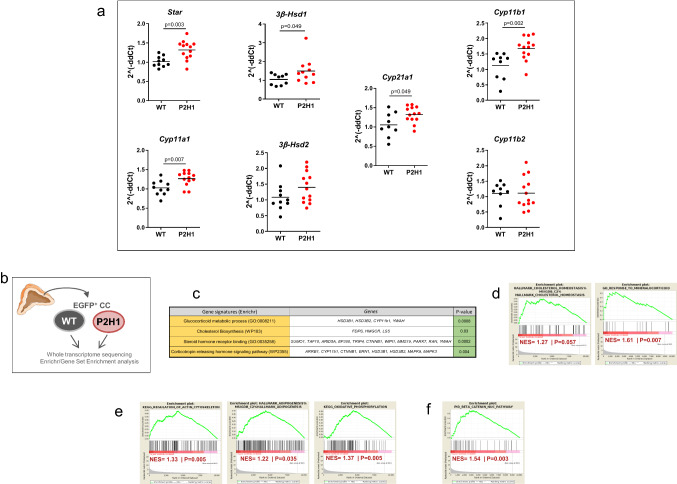
Gene expression analysis of P2H1 adrenocortical cells. **a** Gene expression analysis of enzymes involved in the steroidogenesis pathway using mRNA from whole adrenals from P2H1 mice and WT counterparts (*n* = 10–13). All graphs are the result of 2 independent experiments. **b** Schematic overview of the RNAseq approach which compared sorted GFP^+^ cells from WT controls and P2H1 mice (*n* = 3). **c** Gene signature analysis using Enrichr. **d** Gene set enrichment analyses (GSEA) showed positive signatures for steroidogenesis-related pathways. **e** Prominent HIF-related pathways. **f** The β-catenin nuclear pathway

Previous in vitro studies and reports on HIF1α alterations in zebrafish larvae have suggested negative regulation of Star, the mitochondrial cholesterol transporter [7, 19, 22]. However, data on the effects of HPP alterations in adrenal cortex of mice is scant at best. Therefore, to assess the impact of HIF1α-deletion and/or HIF2α-upregulation in adrenal cortical cells, we performed broad transcription analysis of proteins/enzymes involved in steroidogenesis using mRNA from whole adrenals. Our results reveal that almost all of the gene products tested showed either a significant increase or a tendency to do so, including key enzymes such as *Star*,* Cyp11a1*,* Cyp21a1* and *Cyp11b1* (Fig. 3a).

To further characterize this phenotype driven by the HPPs, we performed *next-generation sequencing* (NGS) and compared the steady-state transcriptomes of P2H1 and WT littermate mice (Fig. 3b). For this, we specifically created the *Akr1b7:cre-PHD2/HIF1/mTmG*^fff/fff^ mouse line (P2H1 reporter mice) to study only targeted adrenal cortex cells, with *Akr1b7:cre-mTmG*^f/f^ animals used as controls. Bulk RNAseq was performed on GFP^+^-sorted adrenal gland cells as described previously [42] and gene signatures of the various lineages were evaluated using Enrichr or gene set enrichment analyses (GSEA). Concurring with the previous results, we found a number of significant signatures related to the process of steroid synthesis in adrenocortical cells or their response to it (Fig. 3c-d). Notably, GSEA also revealed known HIF-dependent associations including, actin cytoskeleton [43, 44], adipogenesis [45] and oxidative phosphorylation [46] (Fig. 3e). Furthermore, P2H1 cortical cells also displayed a positive signature related to the regulation of nuclear β-catenin signaling, which is known to be primarily activated in the zona glomerulosa with potential hyperplasic effects [47] (Fig. 3f). Thus, hypoxia pathway proteins are directly involved in the regulation of known signaling pathways driving adrenal gland homeostasis.

### Modulated adrenocortical HIFs skew cytokine production and leukocyte numbers

**Fig. 4:**
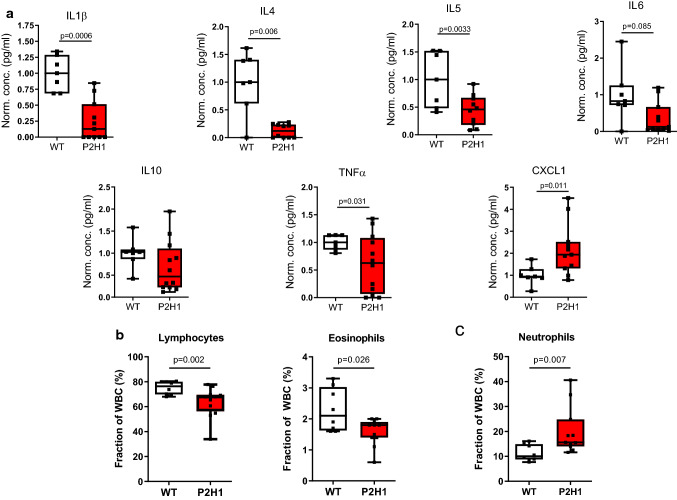
Immune system changes in P2H1 mice. **a** Box and whisker plots representing levels of pro/anti-inflammatory cytokines measured in the plasma of P2H1 mice and WT littermate controls (n = 7–12). All data were normalized to the average value seen in WT mice. Each dot represents data from one animal. **b** Box and whisker plots showing percentage lymphocytes and eosinophils in circulation which revealed reduced fractions in P2H1 mice compared to WT controls. **c** Greater numbers of circulating neutrophils in P2H1 mice compared to WT littermates. All graphs represent pooled results of 2 independent experiments

As several studies have reiterated a crucial role for glucocorticoids in immunomodulation [3, 48], and Cushing’s syndrome has been described to be accompanied by immune deficiency [3, 49, 50], we measured circulatory cytokine levels. We report a substantial overall decrease in the levels of both pro- and anti-inflammatory cytokines, with the exception of the chemokine and neutrophil attractant CXCL1, which increased almost twofold (Fig. 4a). Glucocorticoids have been repeatedly shown to promote apoptosis-mediated reduction of lymphocytes [51] and eosinophil reduction [52], along with neutrophilia due to enhanced recruitment from the bone marrow [53]. Therefore, we enumerated the various white blood cell (WBC) fractions in P2H1 mice and compared it with that of their WT littermates, which revealed a significant reduction in both lymphocyte and eosinophil fractions (Fig. 4b) accompanied by marked elevation in neutrophils (> 70% compared to WT) (Fig. 4c). Taken together, our data reveal a critical role for HIFs in steady-state cytokine levels and leukocyte numbers, probably through alterations in steroidogenesis pathways.

### HIF1α inversely regulates steroidogenesis

**Fig. 5:**
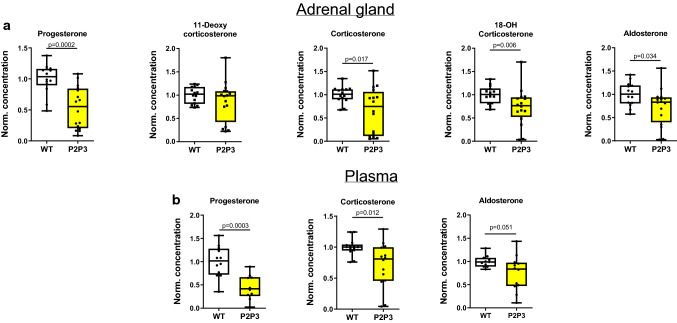
Adrenal cortex-specific loss of PHD2 and PHD3 leads to reduced steroidogenesis in mice. **a** Box and whisker plots showing steroid hormone levels in the adrenal glands of WT mice and compared to that of littermate P2H1 mice (*n* = 14–16 individual adrenal glands). **b** Box and whisker plots showing steroid hormone measurements in the plasma of individual mice (*n* = 10–12). All data were normalized to the average value of WT mice and graphs represent results of pooled data of at least 3 independent experiments

**Fig. 6:**
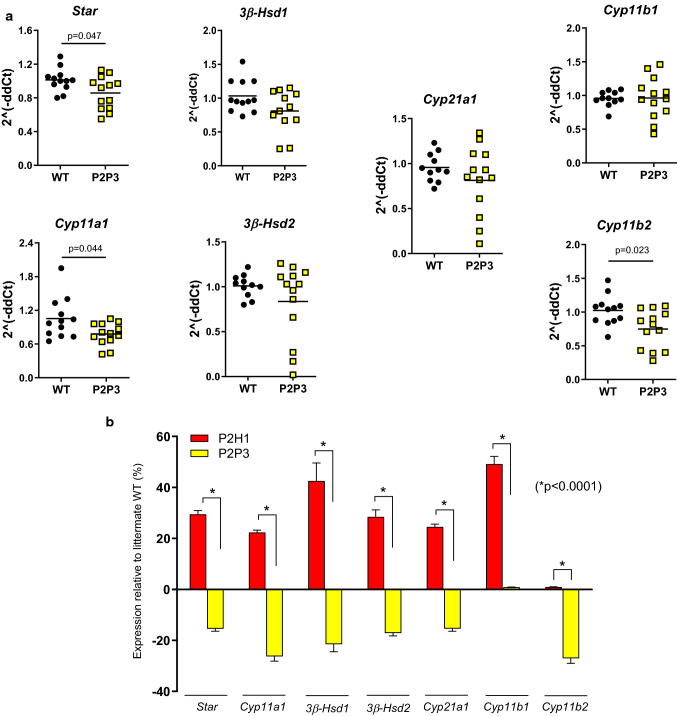
Inverse regulation of steroidogenesis in P2P3 mice compared to P2H1 mice. **a** Gene expression analysis of enzymes involved in the steroidogenesis pathway in P2P3 mice and their WT counterparts (*n* = 12–13) was performed on mRNA from entire adrenal glands. All graphs represent pooled data from at least 3 independent experiments. **b** Relative expression profile of all genes analyzed from the adrenal glands of P2H1 and P2P3 mice and compared to their respective WT littermates. Statistical significance was defined using an unpaired multiple *t* test (*n* = 13; Benjamini, Krieger and Yekutieli method; *p < 0.0001 for all individual genes)

**Fig. 7:**
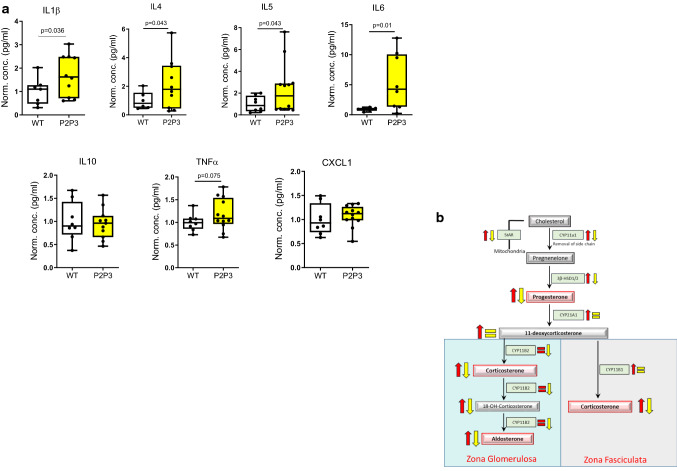
Immune system changes in P2P3 mice. **a** Box and whisker plots representing levels of pro/anti-inflammatory cytokines measured in the plasma of P2P3 mice and WT littermate controls (*n* = 6–13). Each dot represents data from one animal. All data were normalized to the average value seen in WT mice and one-tailed hypothesis tests were performed. All graphs represent samples of different litters. **b** Schematic overview of all changes in adrenocortical enzymes and their corresponding hormones and intermediates reported here in P2H1 (red) and P2P3 (yellow) mice

To extend our understanding of the role of HIF1α and/or HIF2α in adrenocortical cells, we created the Akr1b7:cre-PHD2/PHD3^ff/ff^ mouse line (designated as P2P3) to locally increase both HIF1α and HIF2α (Supplementary Fig. S2). Intriguingly and in contrast to hormone levels in the adrenal glands of the P2H1 mice, P2P3 adrenal glands displayed a marked decrease in corticosterone and aldosterone levels, along with a cognate reduction in their precursors, both in the adrenal gland (Fig. 5a) and in circulation (Fig. 5b). These results clearly suggest that steroidogenesis is dependent on HIF1α but not HIF2α. To further confirm this observation, we performed mRNA expression analyses to identify the levels of central enzymes, similar to that performed in P2H1 mice, and demonstrate an overall decrease in these enzymes (Fig. 6a). This observation is contrary to that seen in the P2H1 mice but fits neatly with the observed reduction in steroid levels in the P2P3 mice, thereby underscoring the central role of HIF1α (Fig. 6b). Finally, we tested the same set of pro- and anti-inflammatory cytokines as for P2H1 mice. In line with all previous results, we show an overall increase in cytokines in the plasma of P2P3 mice versus their WT littermate controls (Fig. 7a). However, no changes were found in CXCL1 protein, which was also reflected in the lack of difference in circulating neutrophils (Supplementary Fig. S3).

## Discussion

Here, using a unique collection of adrenocortical-specific transgenic mouse lines, we identify HIF1α as a central transcription factor that regulates the steroidogenesis pathway by regulating key enzymes. Notably, this directly modifies the entire spectrum of steroid hormones, both in the adrenal gland and in circulation, which eventually impacts the availability of a variety of cytokines.

Studies on the role of HIFs in the regulation of steroidogenesis in vivo are few, apart from those in zebra fish larvae that describe differential regulation of the enzymes involved in the steroid pathway [7, 20, 22]. However, to the best of our knowledge, there are no mouse models to study the role of HPPs in adrenal cortical cells. Undoubtedly, such models would help us to better understand the crosstalk between HPPs and adrenal steroid metabolism, while simultaneously serving as an essential tool to study the pathophysiology of multiple conditions associated with dramatically altered steroid hormone levels [2]. Ablation of HIF1α revealed an important role for this transcription factor in steroidogenesis, which concurs with results from previous studies [22, 54]. Our findings that HIF1α deletion results in the upregulation of mRNA of a vast majority of steroid-related enzymes appear counterintuitive. However, Wang and colleagues recently described three HIF1-binding sites (HREs) in the promoter region of *Star* and a negative regulatory effect of HIF1 on *Star* transcription and synthesis [19]. Furthermore, a number of putative HREs are predicted in the promoter of other steroidogenic enzymes (e.g., in *Cyp11b2* (D.W., A.S and B.W. unpublished results). In-depth analysis of these regions is, therefore, essential to better comprehend the direct negative regulation of steroidogenesis-related enzymes by HIF1. On the other hand, also an indirect effect with potential involvement of one or more transcriptional repressors could play a role [55, 56]. This type of transcriptional regulation of adrenal steroidogenesis has already been suggested with miRNAs, some of which might be directly regulated by hypoxia/HIF1 [57, 58]. Taken together, more in-depth studies are required to completely understand the direct or indirect impact of HIF1α on the expression patterns of steroidogenesis-related enzymes.

Our RNAseq analysis of Akr1b7^+^ P2H1 adrenocortical cells not only unearthed several genetic signatures directly associated with steroidogenesis, but a number of GSEAs revealed prominent HIF-dependent phenotypes previously identified in a variety of other cell types. Recently, we have described a significant role for HIF2α in the regulation of the actin cytoskeleton, especially in facilitating enhanced neutrophil migration through very confined environments [44], HIF1α has also been associated with cytoskeleton structure and functionality in a number of cell lineages (reviewed in [43]); this is apart from its role in energy metabolism wherein enhanced oxidative phosphorylation has been demonstrated in various HIF1α-deficient cell lineages [46]. Therefore, it will be of interest to further explore changes in multiple metabolites that are directly or indirectly associated with the TCA cycle to find a potential link with the overall changes described here.

Glucocorticoids and aldosterone are both essential for homeostasis and their substantial increase in P2H1 mice was intriguing, given their pivotal role in immune suppression [3, 59] and blood pressure regulation, respectively. Previous studies have shown that aldosterone not only increases the expression of the potassium channels that secrete potassium but also stimulates K-absorptive pumps in the renal cortex and medulla, thereby stabilizing and maintaining renal potassium excretion [60], a situation we also observed in the P2H1 mice. The significant increase in glucocorticoids upon HIF1α deletion was clearly associated with immunosuppression, as demonstrated by an overall decrease in both pro- and anti-inflammatory cytokines in circulation, and these observations mirror other reports of immune modulation due to enhanced glucocorticoid levels. Such glucocorticoid elevation can eventually even result in dramatic immune deficiency, for example, as seen in Cushing’s disease [3, 50, 59].

Intriguingly, we found serum CXCL1 to be significantly enhanced in P2H1 mice, probably because as a central neutrophil attractant it was associated with the massive increase in circulatory neutrophils seen in these mice. It is known that enhanced neutrophil recruitment from the bone marrow is directly associated with glucocorticoids [53], as is their overall survival [61].

An essential role of HIF1α, but not HIF2α, in the modulation of enzymes and adrenocortical hormones could be further corroborated by the contrasting results seen in the P2P3 mice. Specifically, compared to P2H1 mice, the expression profile of virtually all steroidogenesis-regulating enzymes was dramatically inverted in the P2P3 mice, which resulted in an overall impairment of the steroidogenesis pathway (Fig. 7b) and an increase in the levels of circulating cytokines. Therefore, these mouse lines will also be helpful to study the potential impact of substantially modulated steroid levels in a variety of clinically relevant diseases including metabolic and auto-immune disorders.

In summary, we reveal a prominent role for HIF1α as a central regulator of steroidogenesis in mice as two distinct transgenic mouse lines showed persistent but contrasting changes in corticosterone and aldosterone concentrations at levels sufficient to modulate systemic cytokine levels and leukocyte numbers. These P2H1 and P2P3 mouse strains will be of significant importance in further exploring the impact of HIF1α in adrenocortical cells and as an important component in regulation of steroidogenesis-mediated systemic effects.

## Supplementary Information

Below is the link to the electronic supplementary material.

Supplementary file1 (DOCX 18 KB)

Supplementary file2 (PDF 39 KB)

## Data Availability

All data and material are available upon request to ben.wielockx@tu-dresden.de.
