# Supplementary material for: HIF1α is a direct regulator of steroidogenesis in the adrenal gland
Source: Cell Mol Life Sci. 2021 Jan 19;78(7):3577–90. doi: 10.1007/s00018-020-03750-1 (PMC8038963; doi:10.1007/s00018-020-03750-1)
Supplement: Supplementary file 1 — Supplementary file1 (DOCX 18 KB) [file 18_2020_3750_MOESM1_ESM.docx]

Online Resource I : Primers for genotyping of mouse strains

| **Primer name** | **Primer sequence (5’ – 3’)** |
| --- | --- |
| Akr1b7_Fw  Akr1b7_Rev | GAAAGCAGGCATTTCATCTGC  CAGGGTGTTATAAGCAATCCC |
| mPHD2_exo2  mPHD2_Intron1 | CGCATCTTCCATCTCCATTT  CTCACTGACCTACGCCGTGT |
| mPHD2_Intron1 | CTCACTGACCTACGCCGTGT |
| mPHD2_Intron3.3 | GGCAGTGATAACAGGTGCAA |
| PHD3mFw  PHD3mRev | ATGGCCGCTGTATCACCTGTAT  CCACGTTAACTCTAGAGCCACTGA |
| PHD3Rec55  PHD3mouseRev | CTCAGACCCCCTAAGTATGT  CCACGTTAACTCTAGAGCCACTGA |
| HIF1a.For | GCAGTTAAGAGCACTAGTTG |
| HIF1a.Rev | GGAGCTATCTCTCTAGACC |

Online Resource II: Primers for qPCR analysis

| **Primer name** | **Primer sequence (5’ – 3’)** |
| --- | --- |
| StAR_Fwd  StAR_Rev | TCGCTACGTTCAAGCTGTGT  GCTTCCAGTTGAGAACCAAGC |
| Cyb11a1_Fwd  Cyb11a1_Fwd | AGGTCCTTCAATGAGATCCCTT  TCCCTGTAAATGGGGCCATAC |
| 3β_HSD1_Fwd  3β_HSD1_Rev | TGGACAAAGTATTCCGACCAGA  GGCACACTTGCTTGAACACAG |
| 3β_HSD2_Fwd  3β_HSD2_Rev | GGTTTTTGGGGCAGAGGATCA  GGTACTGGGTGTCAAGAATGTCT |
| mCyp21a1_Fwd  mCyp21a1_Rev | AACAGAACCATTGAGGAGGCCTTGA  TCTCCAAAAGTGAGGCAGGAGATGA |
| Cyp11b1_Fwd  Cyp11b1_Rev | CAGATTGTGTTTGTGACGTTGC  CGGTTGAAGTACCATTCTGGC |
| mCYP11b2_Fwd  mCYP11b2_Rev | CAGTGGCATTGTGGCGGAACTAATA  GGTCTGACATGGCCTTCTGAGGATT |
| HIF1α_Fwd  HIF1α_Rev | GGCGAGAACGAGAAGAAAAA  AAGTGGCAACTGATGAGCAA |
| mPHD2_Fwd  mPHD2_Rev | AAGCCCAGTTTGCTGACATT  CTCGCTCATCTGCATCAAAA |
| HIF2α_Fwd  HIF2α_Rev | CTGAGGAAGGAGAAATCCCGT  TGTGTCCGAAGGAAGCTGATG |
| HMOX1_Fwd  HMOX1_Rev | AAGCCGAGAATGCTGAGTTCA  GCCGTGTAGATATGGTACAAGGA |
| BNIP3_Fwd  BNIP3_Rev | TCCTGGGTAGAACTGCACTTC  GCTGGGCATCCAACAGTATTT |
| VEGFA_Fwd | GCACTGGACCCTGGCTTTAC |
| VEGFA_Rev | AACTTGATCACTTCATGGGACTTCT |
