# Supplementary material for: HIF1α is a direct regulator of steroidogenesis in the adrenal gland
Source: Cell Mol Life Sci. 2021 Jan 19;78(7):3577–90. doi: 10.1007/s00018-020-03750-1 (PMC8038963; doi:10.1007/s00018-020-03750-1)
Supplement: Supplementary file 2 — Supplementary file2 (PDF 39 KB) [file 18_2020_3750_MOESM2_ESM.pdf]

Supplementary Figure 1

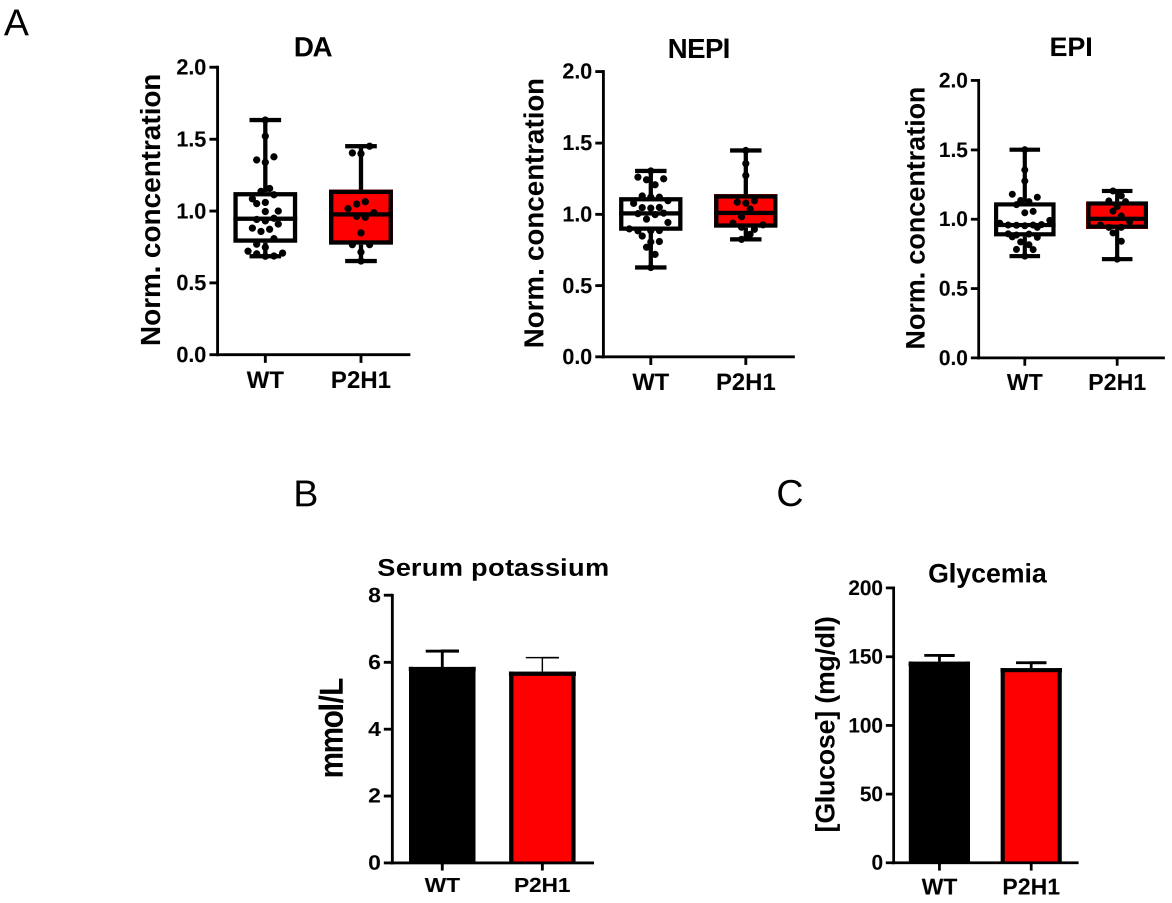

Supplementary figure 1: Downstream effects of increased steroidogenesis.

A. Box and whisker plots showing normalized concentrations of all catecholamines (dopamine, norepinephrine (NEPI), and epinephrine (EPI) measured in entire adrenal glands of P2H1 mice and their WT counterparts (n=14-28). Bar graphs represent, respectively: C. potassium levels in the serum of WT vs P2H1 mice (n=9-11) and D. Blood glucose levels in P2H1 mice vs WT littermate controls (n=5-8). Statistical significance was defined using the Mann-Whitney U test.

Supplementary Figure 2

A

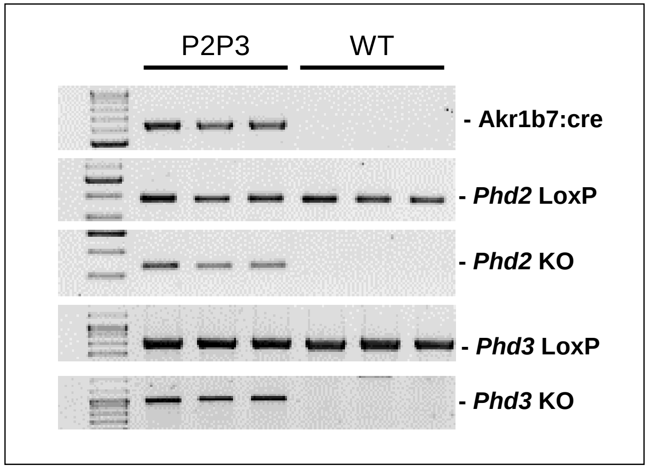

Supplementary figure 2: Genetic identification of the Akr1b7:cre-P2P3 strain. A. Genomic PCRs for Akr1b7:cre, Phd2 LoxP (400bp – fragment encompassing both LoxP sites), Phd2 KO (350 bp – shortened genomic fragment after excision), Phd3 LoxP (840bp - fragment encompassing both LoxP sites) and Phd3 KO (1000bp - shortened genomic fragment after excision) in entire adrenal gland tissue from P2P3 mice and their WT counterparts. 100bp DNA ladder (dense band = 500bp)

Supplementary Figure 3

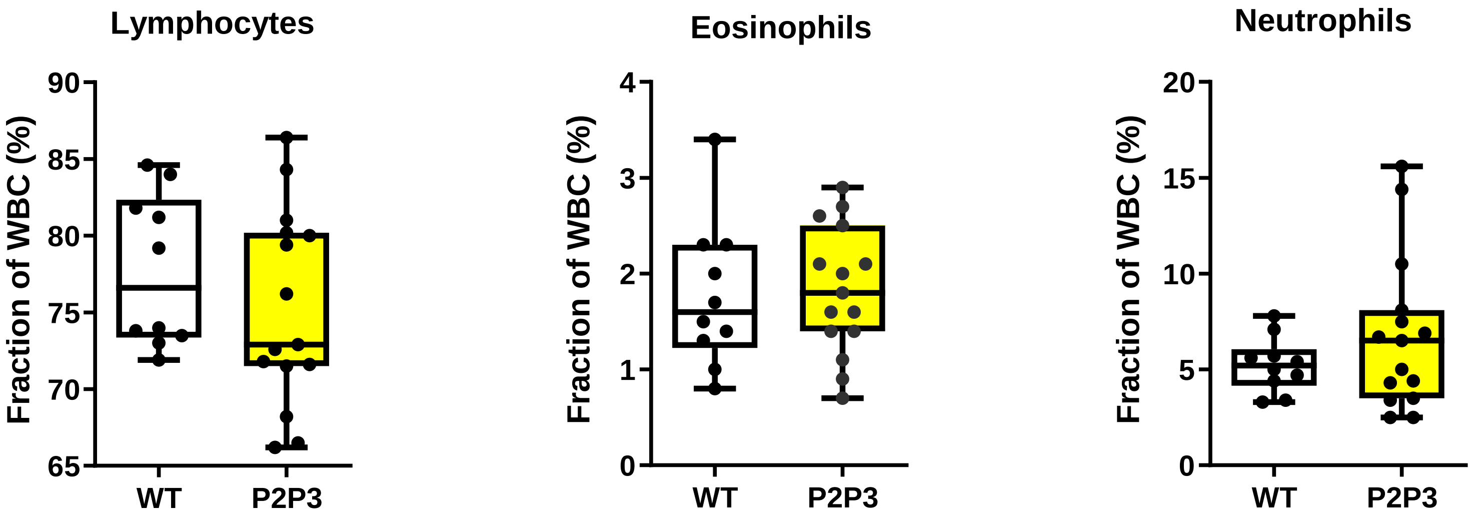

Supplementary Figure 3: Immune cells in circulation in P2P3 mice. Box and whisker plots showing percentage of lymphocytes, eosinophils and neutrophils in circulation. All graphs represent pooled results of >3 independent experiments.
